# A Scoping Review of Interventions for Tobacco Cessation Among African American Individuals

**DOI:** 10.1007/s40429-025-00660-9

**Published:** 2025-05-13

**Authors:** Shubekshya Upadhyay, Abena Duah, Victoria Francois, Sophia I. Allen

**Affiliations:** 1 Department of Public Health Sciences, Penn State Center for Research on Tobacco and Health, Penn State College of Medicine, 500 University Drive, Hershey, PA 17033 USA; 2 Penn State Cancer Institute, 400 University Drive, Hershey, PA 17033 USA

**Keywords:** Smoking cessation, African Americans, Quitlines, Text messaging

## Abstract

**Purpose of Review:**

African American individuals in the US have the highest rates of mortality from diseases such as lung cancer, coronary heart disease, and stroke compared with other minoritized people due to tobacco smoking. Evidence-based interventions are useful for reducing the burden of diseases by helping those who smoke to quit. Despite a higher motivation to quit, African American adult smokers have lower success rates and less access to evidence-based interventions. Hence, it is important to study the factors associated with unsuccessful quit attempts among this population and to search the literature for gaps that need to be addressed.

**Recent Findings:**

We identified 19 articles that focused on Quitlines and text messaging interventions to help African Americans to quit. The interventions used in the studies were Quitlines, text messaging, telephone counseling and media campaigns. We found that African Americans were more likely to use Quitlines than Whites. Studies indicated that interventions should be tailored according to patient preferences. For example, one of the findings was that participants had mixed feelings about the use of standard and non-standard Quitline services. Individuals aged over 60 years preferred standard services such as telephone counseling and printed materials. However, the younger generation were interested in non-standard services.

**Summary:**

There are only a few studies focused on the use of Quitlines and the utilization of their text messaging service among African Americans. Future studies should focus on the reasons disparities in smoking cessation rates exist among African American individuals and leverage the use of text messaging.

**Supplementary Information:**

The online version contains supplementary material available at 10.1007/s40429-025-00660-9.

## Introduction

Tobacco use is considered the major preventable public health problem in the United States (US) resulting in about 480,000 deaths annually, including deaths from secondhand smoke. The life expectancy among individuals who smoke is about 10 years shorter than those who do not smoke [1]. The risk of premature death from smoking can be reduced by 90% by quitting smoking before the age of 40 [2]. In the US, African Americans have the highest rates of mortality from lung cancer, coronary heart disease and stroke in comparison to other races or ethnicities [3] which are linked to smoking behavior. Consequently, the financial burden due to healthcare costs is disproportionate for African Americans. Evidence-based treatments are encouraged for adults who smoke (AWS) to quit and to reduce relapse rates [4]. However, despite a higher motivation to quit smoking, African American adults have lower success rates of quitting in comparison to White adults [5]. Furthermore, African American AWS who are willing to quit do not use the treatments that are proven to be effective which could increase their chances of succeeding in quitting [4]. Therefore, it is important to understand the reasons behind unsuccessful quit attempts to reduce the burden of disease in African Americans.

The use of mobile phone applications (apps), including text messaging, for quitting smoking have numerous advantages. They are cost effective, easy to use and access, since users can access them in their own environment whenever needed, and the messages are delivered in real time [6]. All states in the US have telephone tobacco cessation services, commonly known as Quitline services at zero cost to the enrollees. These services seem to be better than other services as in person smoking cessation programs have numerous barriers such as higher costs and require travel time to appointments. Quitline services have expanded their reach from 42% in 2008 to 98% in 2021 [6]. The awareness of Quitlines is still low in the general population and AWS think the services provided by Quitlines are only limited to providing telephone-based services [7]. In addition to Quitlines, digital tobacco cessation interventions are also commonly used. Smartphones are ubiquitous which are not just limited to text messages but have the access to videos and graphics which might be useful in smoking cessation [8].

However, it is important to not overlook the traditional methods over the success of these modern interventions. Different studies indicate that young people have greater computer proficiency and are interested in text messages, but those over 60 years still tend to use printed materials. Furthermore, digital inequalities or differences in internet access might increase disparities among minoritized groups [9]. Therefore, understanding the preferences of African American AWS while designing interventions is extremely important [10]. The purpose of this scoping literature review was to summarize the findings of technology interventions for tobacco cessation among African American AWS and to identify the gaps in the literature which may be helpful for enhancing interventions among this population in the future.

### Search Methods

We conducted a review of the literature on smoking cessation interventions guided by the PRISMA extension for scoping reviews [11]. Multiple electronic reference databases PubMed, Google Scholar, and the Cochrane library were searched for keywords such as “Black or African American,” “Smoking Cessation,” “Quitlines,” and “Text messaging.” Table 1 shows the keywords that were searched with the number of articles found. Studies were included if (1) published on or after January 1, 1996, (2) they were peer- reviewed literature, (3) race/ethnicity were included in study, (4) study was conducted in the US, (5) study was on Quitline or text messaging for smoking cessation, (6) included human subjects, (7) subjects were 18 years old or older, (8) current or former AWS, and (9) written in English. Studies were not included if: (1) they were gray literature (editorials, news, letters to editor), (2) published before January 1, 1996, (3) not written in English, (4) studies conducted outside of the US, (5) studies that were not on quitlines/smoking cessation helplines, (6) non-human or animal studies, (7) subjects were less than 18 years old and, (8) participants were never AWS.

**Table 1:** Search strategy

SN	Keyword	Total number of articles
1	Smoking cessation	32,071
2	Text Messaging	4864
3	Black or African American	59,791
4	Quitlines	408
5	Smoking cessation and Black or African American	489
6	Quitlines and Black or African American	8
7	Text messaging and Black or African American	75
8	Smoking cessation and text messaging and Black or African American	4

## Results

**Fig. 1:**
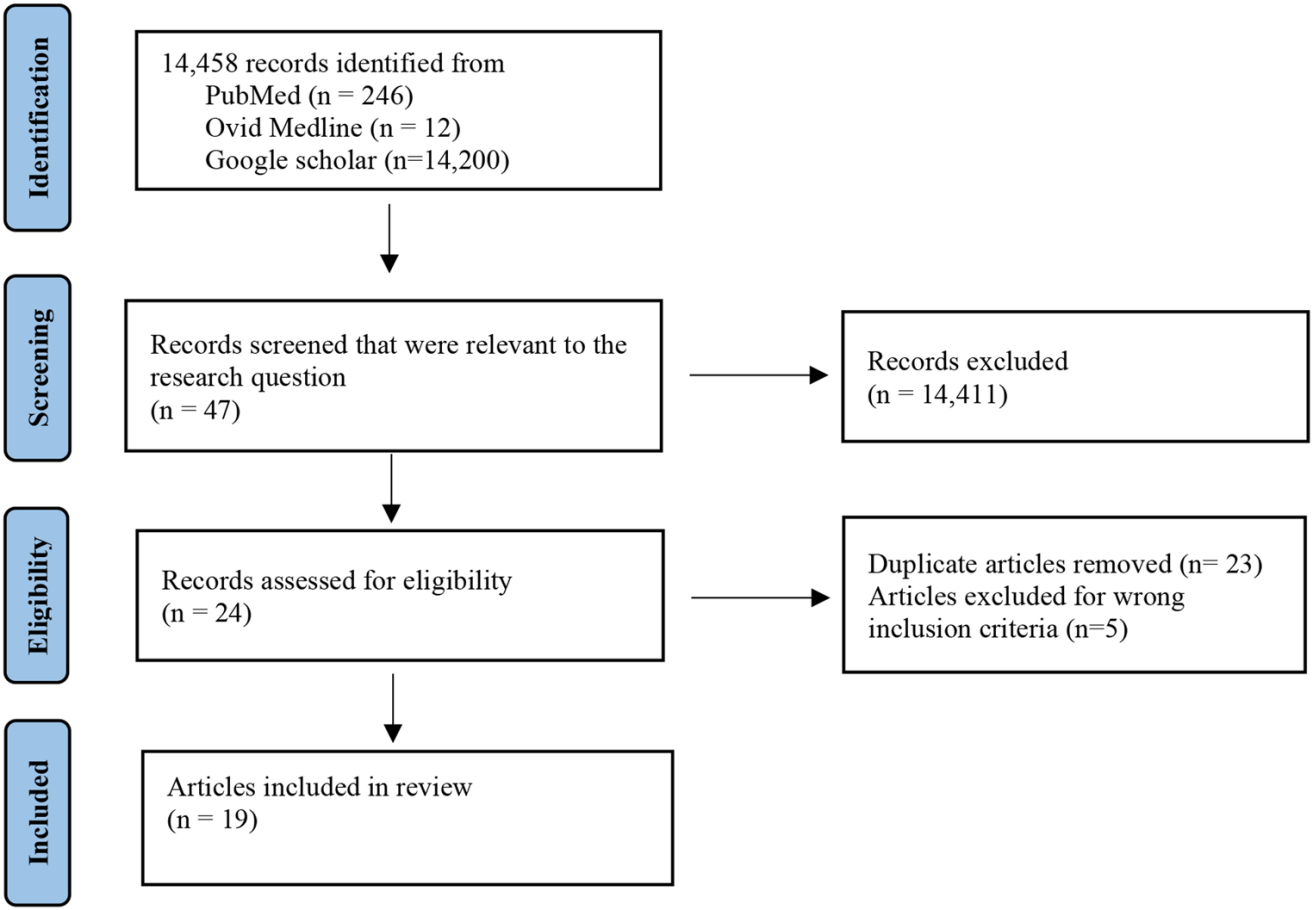
PRISMA flow diagram

The total number of articles found ranged from 408 for the single keyword “Quitlines” to 59,791 for the keyword “Black or African American.” When keyword terms were combined the number of articles found were considerably reduced. After applying the inclusion and exclusion criteria, only 19 articles were relevant and selected for the final review (Fig. 1). Most of the articles in the literature were observational studies and only a few studies were clinical trials. Out of 19 studies, 11 were experimental studies, 4 studies were observational, 2 studies were cross-sectional, 1 study was longitudinal and 1 study was qualitative. An overview of the articles with the author’s name, sample size, intervention, follow up, and outcomes is presented in Table 2. The review is focused on Quitlines and text messaging interventions.

### Summary of Smoking Cessation Interventions

Quitlines are an evidence-based tobacco cessation intervention that offer a variety of free services such as telephone counseling, nicotine replacement therapy (NRT), referral to other resources (behavioral health), and written self-help materials to quit smoking. Mobile health (mHealth) cessation interventions can be administered using mobile phones (smartphones), tablets, and wearables (e.g., FitBit, Apple Watch, etc.). SmokefreeTXT is an SMS text message based smoking cessation program which is free and available nationally in the US. It is a 6–8 week text messaging program that provides texts for smoking cessation tips, cessation motivation, advice on managing cravings, quit smoking facts, and recognition of cessation milestones. A mindfulness based text messaging intervention (iQuit Mindfully) was developed where messages are personalized and interactive. This intervention was developed through an iterative process with African Americans.

### Summary of Studies

Studies were published in peer reviewed journals from 2011 to 2023. A total of 222,834 participants were included in the studies. The study samples were only from the US. The findings from these studies were used to highlight the features of the program which were beneficial for the AWS to quit and the aspects of the programs that could be modified in the future for improvement.

**Table 2:** Description of peer reviewed studies

#	Lead Author	Sample	Study Design	Intervention	Follow up	Outcomes
1	Rabius V. et al. 2012 [23]	• African American-4,483 • Non-Hispanic White-7,328	Experimental Study - RCT	Total participants -3,522 African American- 528 (15%) Telephone interviews were conducted to learn about the effectiveness of quitlines.	7 months	Quitting rates in the group who received only self-help materials were 10% and 12% among African Americans and non-Hispanic Whites, respectively. Quitting rates in the group with counseling were 17% and 21% among African Americans and non-Hispanic Whites, respectively. African Americans requested counseling sessions more than non-Hispanic Whites
2	Bourne DE et al. 2023 [13]	• Total participants-363	Observational Study	N/A	N/A	Quitline serves as the best means to tobacco cessation especially in vulnerable populations.
3	Webb Hooper M et al. 2019 [9]	• Quitline enrollees in 5 states-32,989	Experimental Study	Web only tobacco cessation counseling program included evidence based strategies for tobacco cessation. Tobacco cessation counseling delivered total five telephone counseling sessions.	N/A	African Americans were older than all other groups. African Americans were less likely to get enrolled into web only program through internet. There was digital inequality among the participants.
4.	Kennedy MG et al. 2013 [19]	• Calls were tailored for all callers aged 18 and 45 years and made from counties in the local broadcast range of the radio stations that ran advertisements of Quitlines.	Cohort Study	Campaigns disseminated messages via radio, posters, newspapers and ads on billboards.	N/A	The proportion of calls from pregnant African American women were higher than other races during the campaign.
5	Jenssen BP et al. 2023 [18]	• Total Participants-8,488 • Black/African American-94% • Female-84% • 24–34 years old-56%	Experimental Study	Nicotine replacement therapy, Quitline referral, text message referral	N/A	54% accepted one of the treatment methods
6	Lautner et al. 2018 [21]	• Focus group discussion was done with 21 participants, 10–11 individuals in two groups. • 90% African American • 90% male	Qualitative Study	Focus group discussion focused on questions such as if the participants had heard of quitline.	N/A	There isn’t much information about the smoking cessation strategies so outreach strategies can be developed to help these populations.
7	Monica Webb Hooper et al. 2023 [26]	• 1,053 participants from North Carolina who identified as African American	Experimental Study	Participants were randomized to standard quitline services, quitline services plus Pathways to Freedom (PTF) and quitline services alone.	Baseline, 3- and 6-months post enrollment	The views of PTF were significantly higher than other interventions.
8	Zhu SH et al. 2011 [27]	• African American-61,096 • White-279,042	Observational Study	N/A	N/A	African American smokers used the quitline services more than White smokers.
9	Vijayaraghavan M et al. 2018 [25]	• African American-18,656 • White- 45,907 • English speaking Latinx-12,792 • Spanish speaking Latinx − 3,254	Longitudinal Study	Medical incentives to quit smoking conducted community-based outreach. $20 gift card incentive was given after completion of counseling session and callers were offered free nicotine replacement therapy.	N/A	African American and English speaking Latinx smokers had greater use of the financial incentives than White smokers.
10	Grimes LM et al. 2023 [10]	• Total Participants-1,605 • African American-41% • White-47%	Experimental Study	Standard and non-standard quitline services.	N/A	65% were interested in mobile app, 59% were interested in a personalized web program, and 49% were interested in chatting with online quit coaches.
11	D’Silva J et al. 2019 [16]	Adult smokers enrolled in Minnesota quitline program-10,999	Cross-sectional study	N/A	N/A	Majority of the menthol smokers were female, young, Black/African Americans. State quitlines should focus on allocating resources for menthol users.
12	Spears CA et al. 2019 [24]	Study 1-Initial • Total participants-15 • African American-12 Study 2-Qualitative feedback after text messaging trial • Total participants-10 • African American-8	Experimental Study	Study 1-Focus group discussions were done to provide suggestions for program development. Study 2-One week version of the text messaging program was provided and feedback was obtained through in-depth interviews.	N/A	Participants suggested that the text messaging programs should be personalized and interactive. Participants were using text messages and indicated that the program was useful.
13.	Businelle MS et al. 2016 [14]	• Total participants-59 • African American-53%	Experimental Study	Standard smoking cessation care (group counseling and smoking pharmacotherapy) and smartphone with a novel smoking cessation app.	13 weeks	The intervention was found to be useful for smoking cessation and had positive feedback from the participants.
14	Robinson CD et al. 2020 [22]	• Black-1,333 • White-7,155	Observational Study	Participants were involved in 6 weeks smoke free text program between August 2017 and June 2018. The association between race and program completion was assessed.	6 weeks	Blacks were more dependent on nicotine than Whites, Blacks were more likely to complete the program than Whites, despite having started the program at a similar frequency. Self reported abstinence was lower among Blacks for all seven smoking assessments.
15	Graham A et al. 2021 [17]	• Total participants-618 • African American-109 • White-435	Experimental Study- RCT	Participants were divided into treatment arms and had access to both website and text messaging; controls had access to only the website.	9 months	The abstinence rate among the treatment group was 23.1% and 23.2% among the control group. The effectiveness of website and text messaging combined was similar to the website alone.
16	Kathuriya H et al. 2022 [20]	• Total participants-25 • African American-36% • Unemployed − 92%	Experimental Study	28-day SMS text messaging program. Provided smoking cessation support. Participants were enrolled into two groups: ready to quit within 30 days and not ready to quit within 30 days. The automated text messages were sent two times daily for four weeks. Text messages focused on topics such as health and cost benefits of quitting, managing stress and mood, motivation, coping strategies and encouragement, and addressing medication misconceptions.	30 days	84% of the participants activated the program and none of them unsubscribed. People perceived that the text messaging program motivated them to stop smoking.
17	Cottrell-Daniels C et al. 2021 [15]	• Total participants-32 • African American-90.6%	Experimental Study	iQuit Mindfully, personalized interactive text messaging program; semi-structured interviews	8 weeks	The text messaging program was feasible and acceptable to low-income African Americans. Participants felt that personalized texts and momentary support might help in smoking cessation.
18	Shuter J et al. 2020 [8]	• Total participants-100	Experimental Study-RCT	48 participants were randomized to positive smoke free mobile (81% Black, 31% Latino) and 52 participants were randomized to standard care.	3 months	Abstinence at 3 months, quit attempts and lower intake of cigarettes were significantly higher in positive smoke free mobile (PSF-M) group rather than standard care. 61% of the participants mentioned that PSF-M was extremely helpful and 98% said that they would recommend it to friends or family.
19	Alcaraz, KI et al. 2018 [12]	• Total participants-15 African American socioeconomically disadvantaged	Cross-sectional mixed method study	N/A	N/A	Smartphone ownership was high; however, the use of smartphones for smoking cessation resources was low. Participants preferred email messages over text messages for smoking cessation.

*Notes*: N/A = not applicable: Information was not included in the study

## Discussion

This scoping review examined the use of Quitlines and text messaging for smoking cessation interventions in the last decade among African American AWS. Unfortunately, not much has changed as few studies focused research exclusively on this specific population. Even more dire is the lack of clinical trials on smoking cessation among African American AWS, who are more likely to die from smoking than other groups.

We found that African American AWS were more likely to use Quitlines than White AWS. This could be due to Quitlines being convenient, cost effective and semi-anonymous. The majority of participants who were younger mentioned that they preferred non-standard Quitline services such as a mobile app, personalized text messages or personalized web programs over traditional services such as nicotine replacement therapy and a phone call with the counselor [7]. This study also emphasized that people over 60 years old were more interested in standard Quitline services such as telephone counseling and printed materials and younger people along with people who have low incomes were attracted to mobile apps and personalized web-based programs. This indicates that future interventions should focus on participant preferences, a point also supported by Kathuria and colleagues [20], who noted that individuals have varied reactions to different topics in text messages. For instance, messages that focused on cost and health benefits were highly accepted whereas those discussing how quitting smoking might help prevent relapse into other substances had mixed views. It was also evident that adding text messages to other interventions did not increase smoking abstinence.

Similar to the findings of Spears and colleagues [24] this study reported that interactive and personalized text messaging programs were suggested by the participants for better results. In addition, participants mentioned that individualizing the timing and the number of text messages would be helpful in supporting their autonomy [16]. However, in contrast to this, according to Alcaraz and colleagues [12] the majority of the participants preferred emails over text messaging. Emails were most likely to be more appealing and convenient than text messaging especially for AWS and those who are socioeconomically disadvantaged. Graham and colleagues [18] reported that the abstinence rates among participants with combined interventions of text messaging and the internet were similar to the abstinence rates among participants with an internet only intervention group.

Rabius and colleagues [23] reported that telephone counseling was effective in reducing the tobacco cessation rates among African American AWS, which was similar to non-Hispanic White AWS. Similar to this finding, Robinson and colleagues [22] mentioned that Black participants were equally likely to remain in text messaging programs as Whites. The higher enrollment rates of Black participants indicated their willingness to quit smoking. Despite their higher enrollment, their abstinence rates were lower. There might be several possible reasons behind it, (1) they might not be opting out of the program but using it passively so not benefiting, and/or (2) they might not be interested in providing the information about their quitting which could result in inaccurate documentation. A recent study by Budenz and colleagues [28] showed that people who had not responded had already quit smoking.

Research shows that higher engagement aids in abstinence from smoking so future interventions should focus on ways to increase engagement. The meta-analysis conducted by Scott and colleagues [6] indicated that a text messaging intervention was extremely beneficial for smoking cessation and no further RCTs are necessary to assess the efficacy of such interventions for smoking cessation. Webb Hooper and colleagues [9] reported that participants preferred telephone counseling rather than web-based coaching. The increasing digital interventions are cost effective and might be convenient to target younger smokers; however, digital tobacco cessation programs might create disparities as minoritized groups are more comfortable with the traditional approach.

## Limitations

The current study has certain limitations. Our study is cross-sectional, hence, we are not able to analyze behavior of smokers over a period of time as this study captures the snapshot of articles published at a specific point in time. The study is limited to the use of Quitlines and text messaging interventions for smoking cessation by African American AWS and cannot be generalized to another race or population group. Also, the studies reviewed were conducted in the US and are not generalizable globally. Furthermore, most of the studies in the review were observational studies and only a few were clinical trials. Since observational studies are not as strong as clinical trials, the results might be subjected to bias and confounding. We also emphasized that there hasn’t been much research on user characteristics that are linked to engagement with smartphone or web-based interventions among African American AWS. While a systematic review may provide a more comprehensive evaluation of the literature, we have confidence that due to the lack of studies exclusively focused on African Americans, our review of articles with studies focused in the US has provided a basis on which to build future research.

## Conclusion

This scoping review indicated that there has not been much research on the use of Quitlines or text messaging among African American AWS thus demanding for more studies in the future. Qualitative and mixed methods studies may be required to better understand the perspectives of this specific population. Additionally, unique, culturally specific and tailored interventions should be implemented that consider the challenges with quitting menthol cigarettes and coping with stress caused by social determinants, including systematic racism. Fortunately, several federally funded studies are currently in progress to address the disparities in smoking cessation among African American AWS [29].

## Key References

- Hooper MW, Carpenter KM, Salmon EE, Resnicow K. Enhancing tobacco quitline outcomes for African American adults: an RCT of a culturally specific intervention. American Journal of Preventive Medicine. 2023 Dec 1;65(6):964 − 72. 10.1016/j.amepre.2023.06.005.
-
  - This study tested the effectiveness of a culturally specific tobacco cessation video intervention among African American quitline enrollees.
- Graham AL, Papandonatos GD, Cha S, Amato MS, Jacobs MA, Cohn AM, Abroms LC, & Whittaker R. Effectiveness of an optimized text message and internet intervention for smoking cessation: A randomized controlled trial. Addiction (Abingdon, England), 2022;117(4), 1035–1046. 10.1111/add.1567.
-
  - A parallel two group individually randomized controlled trial where participants were divided into treatment arms who had access to both website and text messaging and control arms who had access only to the website.
- Grimes LM, Garg R, Weng O, Wolff JM, McQueen A, Carpenter KM, et al. Appeal of tobacco Quitline services among low-income smokers. Preventing Chronic Disease. 2023;20. 10.5888/pcd20.220214.
-
  - Participants were from 13 tobacco Quitline services who had called the helpline number. The services were classified as standard services (call from coach, nicotine replacement therapy, printed cessation booklets) or non-standard services (mobile app, personalized text, personalized web and online chat with coach).

## Electronic Supplementary Material

Below is the link to the electronic supplementary material.

Supplementary Material 1

## Data Availability

No datasets were generated or analysed during the current study.
